# ﻿*Chasitermespax*, a new genus and species of soldierless termite (Termitidae, Apicotermitinae) from the island of Trinidad

**DOI:** 10.3897/zookeys.1139.94972

**Published:** 2023-01-11

**Authors:** Rudolf H. Scheffrahn, Tiago F. Carrijo

**Affiliations:** 1 Fort Lauderdale Research and Education Center, Institute for Food and Agricultural Sciences, University of Florida, 3205 College Avenue, Davie, Florida 33314, USA University of Florida Davie United States of America; 2 Centro de Ciências Naturais e Humanas, Universidade Federal do ABC, Rua Arcturus 03, Jardim Antares, 09606-070, São Bernardo do Campo, SP, Brazil Universidade Federal do ABC São Bernardo do Campo Brazil

**Keywords:** *Anoplotermes*-group, enteric valve, Isoptera, Neotropics, new species, taxonomy

## Abstract

*Chasitermespax* Scheffrahn & Carrijo **gen. et sp. nov.** is described from workers collected from a single colony in the Northern Range of Trinidad. The shape and texture of the unsclerotized enteric valve, tubular shape of the enteric valve seating, and prominent spherical mesenteric tongue of *C.pax* are the diagnostic characters for both the genus and species. A Bayesian phylogenetic analysis using the COI gene and including all neotropical Apicotermitinae genera described to date supports the new genus as a distinct terminal.

## ﻿Introduction

The soldierless termites of the New World form a monophyletic clade (Romero Arias et al. 2021) that comprises 16–47% of the termite diversity in Amazonian ecosystems (Bourguignon et al. 2016b). Although the richness of soldierless taxa is recognized, most have not yet been described (Bourguignon et al. 2016b). Originally, all neotropical soldierless termites were placed in the genus *Anoplotermes* Müller, 1873. Recognition of much greater taxonomic diversity began with Mathews (1977) who described *Grigiotermes* and *Ruptitermes*, and Fontes (1986) who described *Aparatermes* and *Tetimatermes*. Fontes (1992) provided the first identification key for workers of these five genera. The descriptions of *Longustitermes* (Bourguignon et al. 2010), *Compositermes* (Scheffrahn 2013), *Amplucrutermes*, *Humutermes*, *Hydrecotermes*, *Patawatermes*, and *Rubeotermes* (Bourguignon et al. 2016a), *Disjunctitermes* (Scheffrahn et al. 2017), *Echinotermes* and *Rustitermes* (Castro et al. 2018, 2020, respectively) and, for now, *Tonsuritermes* and *Dissimulitermes* (Constantini et al. 2018, 2020, respectively) have expanded the defined diversity of neotropical soldierless taxa.

Trinidad and Tobago are continental islands that separated from Venezuela during the Holocene (Comeau 1992; Mychajliw et al. 2020). As such, they have a rich diversity of Amazonian flora and fauna. The University of Florida Termite Collection (UFTC) database shows that 24 described and undescribed genera of Apicotermitinae occur on the islands (Scheffrahn 2019); about the same number as northern Venezuela (Scheffrahn 2019) and possibly somewhat more in French Guiana (Davies 2002; Bourguignon et al. 2013).

In this paper we describe *Chasitermespax* gen. et sp. nov. based on the morphology of the worker caste and molecular data.

## ﻿Materials and methods

Workers were collected and preserved in 85% ethanol. External and internal dissections were suspended in Purell Instant Hand Sanitizer in a plastic Petri dish and photographed using a Leica M205C stereomicroscope controlled by Leica Application Suite ver. 3.0 montage software. The enteric valve (EV) was prepared by removing the entire worker proctodeal segment (P2) section in ethanol. Food particles were expelled from the P2 tube by pressure manipulation. The tube was quickly submerged in a droplet of PVA medium (BioQuip Products Inc.) which, by further manipulation, eased muscle detachment. The remaining EV cuticle was left intact or longitudinally cut, splayed open, and mounted on a microscope slide using the PVA medium. The EV was photographed with a Leica CTR 5500 compound microscope with phase-contrast optics using the same montage software. Terminology of the worker gut follows that of Sands (1972) and Noirot (2001). Mandible terminology as in Sands (1972) except for the left subsidiary fourth marginal tooth which was clarified and redefined as the “premolar process” (Constantini et al. 2020).

The barcode region of the mitochondrial gene cytochrome c Oxidase subunit I (COI) of *Chasitermespax* was obtained by DNA extraction and PCR performed by the Canadian Centre for DNA Barcoding (BOLD systems) following standard high-throughput protocols (deWaard et al. 2008). The PCR employed the primers *LepF1* and *LepR1* (Hebert et al. 2003) which generated 658bp. A gene tree was created under Bayesian Inference (BI) using the COI. In addition to the sequence of *C.pax*, a total of 56 GenBank or BOLD sequences were used: 36 sequences of neotropical Apicotermitinae (23 species, 15 genera), eight Old World Apicotermitinae genera, five non-ApicotermitinaeTermitidae, and one Rhinotermitidae, [*Heterotermescrinitus* (Emerson)] as the outgroup. Sequences were aligned under MUSCLE algorithm implemented in Geneious ver. 9.1.8 (Biomatters Ltd., Auckland, New Zealand). Substitution model used (GTR+I+G) was selected through the Akaike Information Criterion (AIC) with the software jModelTest2 (Darriba et al. 2012). The XML input files were generated with BEAUti ver. 1.8.0, and the BI was performed with BEAST ver. 1.8.0 (Drummond et al. 2012). A Yule speciation process was used as the tree prior. Three Markov chain Monte Carlo (MCMC) searches were conducted independently, each one for 20,000,000 generations, and they were combined to search the most probable tree. Convergence and stationarity were assessed with Tracer ver. 1.5 (Rambaut et al. 2014) and the first 100 trees were discarded as burn-in with TreeAnnotator ver. 1.8.0 and visualized using FigTree ver. 1.3.1.

## ﻿Taxonomy

### ﻿ Chasitermes

Taxon classificationAnimaliaBlattodea Termitidae

Scheffrahn & Carrijo gen. nov.

B9DFDB0D-EC38-5B23-A0F8-1060DF188C1F

https://zoobank.org/84F91A07-5829-4250-84EB-305E9BCFE300

#### Type-species.

*Chasitermespax* Scheffrahn & Carrijo, sp. nov.

#### Diagnosis.

The combination of unsclerotized rectangular EVA cushions, a tubular extension of EVS, and a prominent spherical mesenteric tongue make *C.pax* unique among all Apicotermitinae genera.

#### Description.

**Figure 1.**
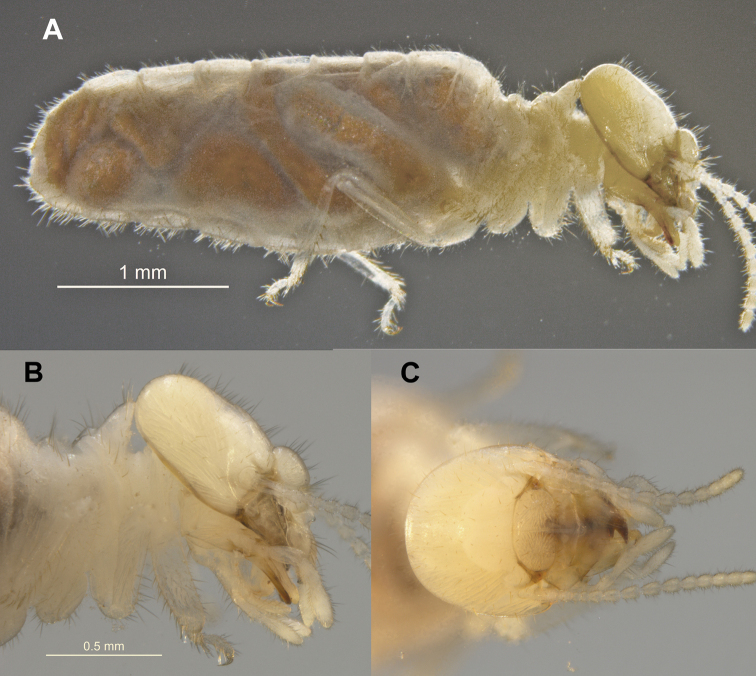
*Chasitermespax* gen. et sp. nov. **A** right lateral habitus of worker **B** lateral and **C** dorsal views of head.

**Figure 2.**
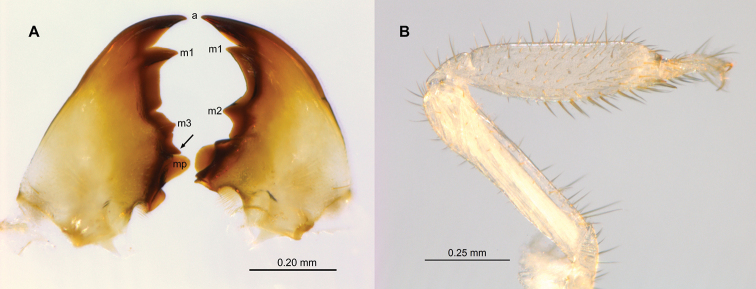
*Chasitermespax* gen. et sp. nov. **A** mandibles (abbreviations: a = apical, m1-m3 = marginal teeth, mp = molar process, arrow = premolar process (Constantini *et al.* 2020) **B** right fore tibia.

**Figure 3.**
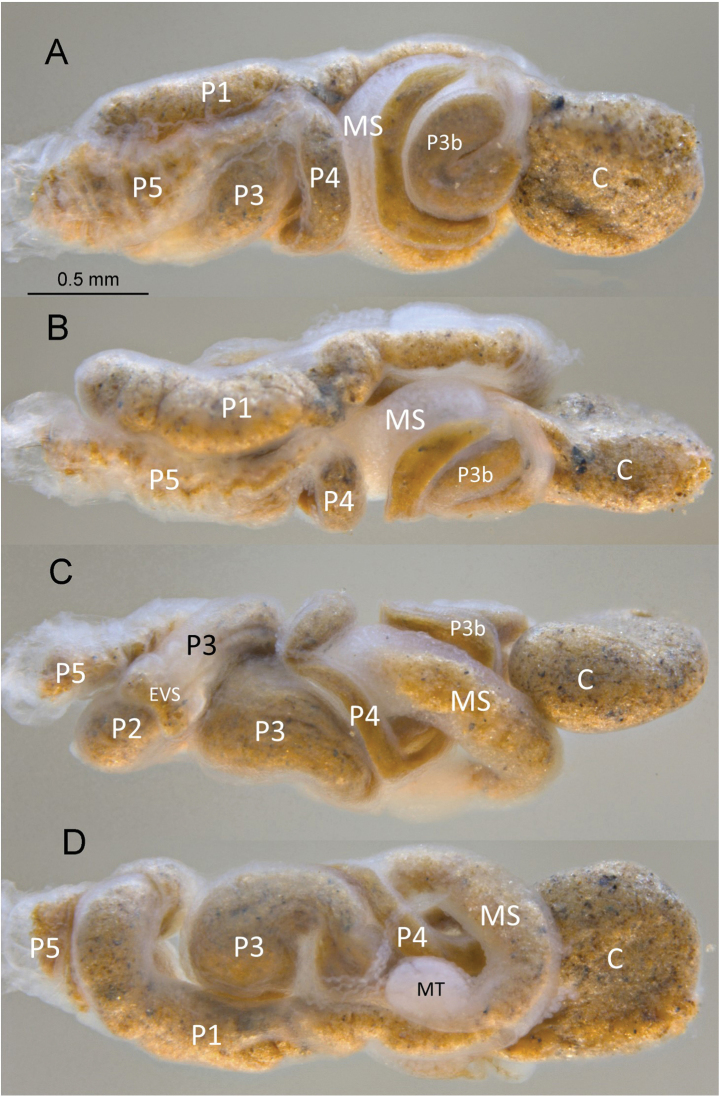
*Chasitermespax* gen. et sp. nov. digestive tube **A** dorsal **B** right **C** left, and **D** ventral (abbreviations: C = crop, EVS = enteric valve seating, MS = mesenteron, MT = mesenteric tongue, P1–P5 = proctodeal segments).

**Figure 4.**
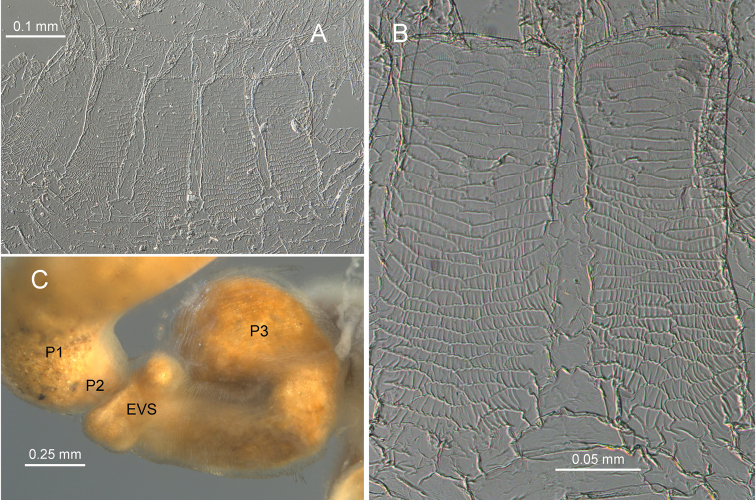
*Chasitermespax* gen. et sp. nov. **A** spliced whole mount of enteric valve armature (rightmost cushion torn) **B** close-up of two cushions **C** region encompassing EVS (abbreviations as in Fig. 3).

Imago unknown. Worker. (Figs 1–4). Monomorphic. Head capsule and antennae light yellowish. Head covered with ca 50 longer (0.1 mm) setae and a few shorter ones (Fig. 1). In lateral view, dorsal surface of the head capsule slightly convex; postclypeus is moderately inflated. Antennae with 14 articles (formula 2>3≈4<5). Pronotum with about 20 long setae concentrated mainly at the borders and a few shorter ones. Mandibles with apical teeth very prominent; left mandible with M1 triangulate, shorter than apical; premolar process longer than M3 (Fig. 2A). Apical tooth of right mandible as long as that of left but narrower; M1 as long as M2. Fore tibia moderately inflated (Fig. 2C) with about a dozen thick posterior setae concentrated at the distal half; lateral and anterior surfaces with thinner long and shorter setae. Digestive tube (Fig. 3 with abbreviations) with voluminous crop; mesenteron forming half circle; mixed segment with prominent spherical mesenteric tongue. First proctodeal segment (P1) widens to maximum diameter before P2, enteric valve seating (EVS) slightly trilobed. Third proctodeal segment (P3) with long tubular extension of the EVS seating to main reservoir (Fig. 4C) then narrowing again toward P4 (Fig. 3D). Cuticle of EVA without sclerotization (Fig. 4A, B) forming six rectangular cushions with about 100 scales each (Fig. 4A). Scales proximal to P1 subrectangular, each with 5–10 creases; scales in distal one-third lacking creases, more scale-like with long fringes (Fig. 4B). Measurements are provided in Table 1.

**Table 1.** Measurements (mm, *N* = 10) of *Chasitermespax* gen. et sp. nov. workers.

	Max.	Min.	Mean
Length of head to lateral base of mandibles	0.77	0.70	0.73
Maximum head width	0.93	0.82	0.85
Length of hind tibia	0.77	0.67	0.72
Length of postclypeus	0.25	0.18	0.21
Width of postclypeus	0.44	0.39	0.40
Length of fore tibia	0.67	0.53	0.60
Width of fore tibia	0.18	0.12	0.15
Fore tibia width:length ratio	0.30	0.21	0.25

#### Remarks.

The single most diagnostic character of the *C.pax* worker is the enteric valve armature which has unsclerotized rectangular cushions composed of creased or fringed scales. The tubular extension of the EVS in *C.pax* is closest to *Patawatermesnigripunctatus* (Emerson, 1925) but is much longer in the former and *P.nigripunctatus* lacks a trilobed enteric valve seating. The left mandible of *C.pax* has prominent premolar process closest to *Patawatermesturricola* (Silvestri, 1901) but it is narrower and longer in the former.

#### Etymology.

Named in honor of the collector, James A. Chase.

### ﻿ Chasitermes pax

Taxon classificationAnimaliaBlattodea Termitidae

Scheffrahn & Carrijo sp. nov.

FBA8A963-5C6A-54D8-8857-900EFDCFA56F

https://zoobank.org/3458C43A-3215-49A1-BE7F-A27761032D7D

#### Type locality.

Tunapuna, island of Trinidad.

#### Material examined.

Republic of Trinidad and Tobago, Tunapuna (10.667, -61.396), elev. 248 m, 4JAN2012, J. Chase, UFTC no. TT2188 holotype worker and about 75 additional workers.

#### Diagnosis and description.

As described for the genus.

#### Etymology.

The species is named for the Pax Guest House where we stayed during our expeditions to Trinidad. It is on the tranquil and inspirational property of the Mount Saint Benedict Abby which encompasses the type locality of *C.pax*. “Pax” is latin for “peace”, and represents a noun in apposition.

#### Ecology and distribution.

The *C.pax* workers were collected under a stone. Gut contents confirm that *C.pax* feeds on soil organic matter. So far, this species is only known from the Northern Range on the island of Trinidad.

#### Molecular analysis.

**Figure 5.**
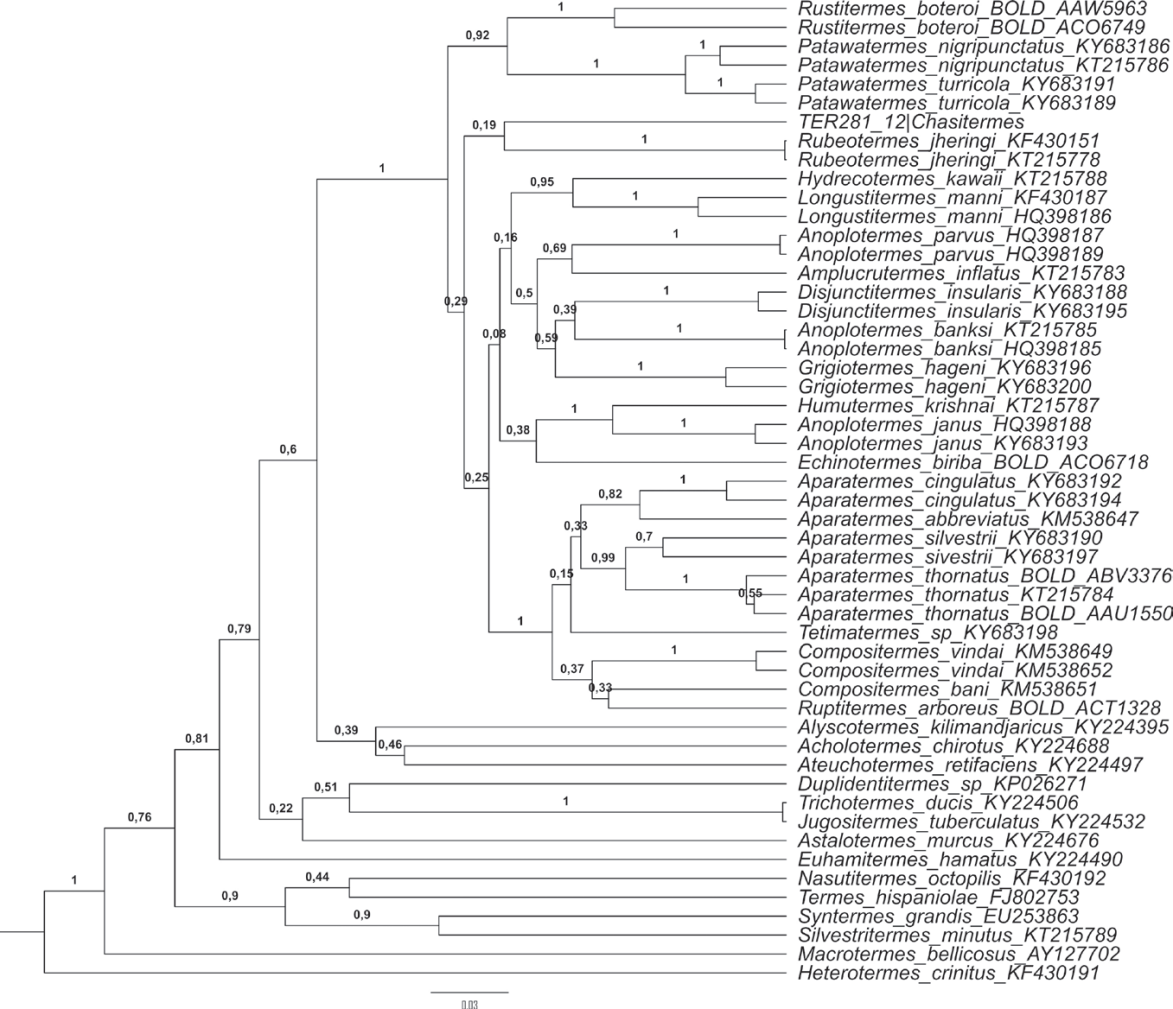
Bayesian phylogenetic tree of the Apicotermitinae subfamily using the COI region. *Chasitermespax* gen. et sp. nov. is shown in red. Branch support is posterior probability.

The gene tree recovered *Chasitermespax* as sister group to *Rubeotermes*, but with very low posterior probability. The low branch support for most major clades in the neotropical Apicotermitinae should be interpreted as a polytomy (Fig. 5).

## ﻿Discussion

Advances in the taxonomy, phylogeny, biogeography, ecology of the Apicotermitinae are ongoing (Bourguignon et al. 2016b, Romero Arias et al. 2021). One area of study that remains poorly understood is the physiology, especially, digestive physiology and its relationship with the gut morphology of these mostly soil-feeding insects. Work by Andres Brune and colleagues (e.g., Schmitt-Wagner and Brune 1999; Brune and Friedrich 2000) provided some insight into the digestive process of the soil-feeding Cubitermitinae. Are there similarities with the Apicotermitinae? What role does such different EVAs, like those of *Chasitermes* gen. nov. and *Patawatermes*, have in the inoculation of the food bolus (Scheffrahn 2013) by the myriad of bacteria (Bourguignon et al. 2018) occurring in the enteric valve seating (suggestively called a “bacterial pouch” by Noirot (2001)? Finally, is the singular P3 shape of *Chasitermespax* related to a different diet or bacterial biota? The present work does not directly contribute to the advances in this particular field, but we hope to instigate terminologists to seek the answers linking the morphology, such as those described here, to the function and biology of the groups.

## Supplementary Material

XML Treatment for Chasitermes

XML Treatment for Chasitermes pax
